# *CDKN2D-WDFY2* Is a Cancer-Specific Fusion Gene Recurrent in High-Grade Serous Ovarian Carcinoma

**DOI:** 10.1371/journal.pgen.1004216

**Published:** 2014-03-27

**Authors:** Kalpana Kannan, Cristian Coarfa, Kimal Rajapakshe, Shannon M. Hawkins, Martin M. Matzuk, Aleksandar Milosavljevic, Laising Yen

**Affiliations:** 1 Department of Pathology & Immunology, Baylor College of Medicine, Houston, Texas, United States of America; 2 Department of Molecular and Cellular Biology, Baylor College of Medicine, Houston, Texas, United States of America; 3 Department of Obstetrics and Gynecology, Baylor College of Medicine, Houston, Texas, United States of America; 4 Department of Molecular and Human Genetics, Baylor College of Medicine, Houston, Texas, United States of America; 5 Department of Pharmacology, Baylor College of Medicine, Houston, Texas, United States of America; 6 Duncan Cancer Center, Baylor College of Medicine, Houston, Texas, United States of America; University of Washington, United States of America

## Abstract

Ovarian cancer is the fifth leading cause of cancer death in women. Almost 70% of ovarian cancer deaths are due to the high-grade serous subtype, which is typically detected only after it has metastasized. Characterization of high-grade serous cancer is further complicated by the significant heterogeneity and genome instability displayed by this cancer. Other than mutations in *TP53*, which is common to many cancers, highly recurrent recombinant events specific to this cancer have yet to be identified. Using high-throughput transcriptome sequencing of seven patient samples combined with experimental validation at DNA, RNA and protein levels, we identified a cancer-specific and inter-chromosomal fusion gene *CDKN2D-WDFY2* that occurs at a frequency of 20% among sixty high-grade serous cancer samples but is absent in non-cancerous ovary and fallopian tube samples. This is the most frequent recombinant event identified so far in high-grade serous cancer implying a major cellular lineage in this highly heterogeneous cancer. In addition, the same fusion transcript was also detected in OV-90, an established high-grade serous type cell line. The genomic breakpoint was identified in intron 1 of CDKN2D and intron 2 of WDFY2 in patient tumor, providing direct evidence that this is a fusion gene. The parental gene, CDKN2D, is a cell-cycle modulator that is also involved in DNA repair, while WDFY2 is known to modulate AKT interactions with its substrates. Transfection of cloned fusion construct led to loss of wildtype CDKN2D and wildtype WDFY2 protein expression, and a gain of a short WDFY2 protein isoform that is presumably under the control of the CDKN2D promoter. The expression of short WDFY2 protein in transfected cells appears to alter the PI3K/AKT pathway that is known to play a role in oncogenesis. *CDKN2D-WDFY2* fusion could be an important molecular signature for understanding and classifying sub-lineages among heterogeneous high-grade serous ovarian carcinomas.

## Author Summary

High-grade serous carcinoma (HG-SC) is the most common subtype of ovarian cancer observed in women. This subtype of ovarian cancer is typically detected at advanced stages due to lack of effective early screening tools. Recurrent cancer-specific gene fusions resulting from chromosomal translocations have the potential to serve as effective screening tools as well as therapeutic targets. Here we identified *CDKN2D-WDFY2* as a cancer-specific fusion gene present in 20% of HG-SC tumors, by far the most frequent gene recombinant event found in this highly heterogeneous disease. We also presented evidence that the expression of this fusion may affect the PI3K/AKT pathway that is important for cancer progression. Thus *CDKN2D-WDFY2* could very well represent a major cellular lineage important for detecting and classifying heterogeneous ovarian carcinomas, and could provide insight into the underlying mechanism of this deadly disease. This is critical, given that ovarian cancer kills 140,200 women worldwide each year, and few ovarian cancer-specific molecular alterations are currently available for targeting and screening.

## Introduction

Ovarian cancer is the most lethal gynecologic malignancy in women. Approximately 225,500 women are diagnosed with ovarian cancer with an estimated 140,200 associated deaths annually [1]. Almost 70% of the ovarian cancer cases are the high-grade serous carcinoma (HG-SC) subtype [1], which is typically detected at advanced stages due to lack of effective screening tools. HG-SC differs substantially from other subtypes of ovarian carcinoma in their molecular features. Common cancer genes such as *TP53* and *BRCA1/2* are mutated in 96% and 22% of HG-SC patients, respectively [2]. These mutations could contribute to the extensive genome rearrangements and high levels of heterogeneity observed in HG-SC [2]. The high degree of heterogeneity in HG-SC suggests diverse clonal lineages within the same patient and among different patients. Discovery of specific molecular signatures for major clonal lineages is essential for understanding the underlying pathogenesis of HG-SC and for designing personalized treatment.

The characteristic massive genome rearrangement in HG-SC implies that recombination events such as gene fusions should be common. If a fusion gene leads to oncogenic consequences, then it will be present in clonal expansions, and therefore, likely recurrent among tumors. Highly frequent gene fusions are significant for several reasons. For example, the *BCR-ABL* fusion gene in chronic myeloid leukemia is known to initiate oncogenesis through the formation and mis-regulation of a fusion protein [3]. The *BCR-ABL* fusion is also a clinical biomarker of high diagnostic and prognostic utility. In addition, this fusion protein serves as a therapeutic target for the drug Gleevec. In prostate cancer, the fusion gene *TMPRSS2-ERG* was found in 50% of patients, and it is used to classify patient groups [4], [5]. Fusion genes of comparable utility and frequency of occurrence are particularly difficult to identify in HG-SC because of the high heterogeneity observed in these tumors. This difficulty is illustrated by a recent study that identified 45 fusion genes in ovarian cancer, none of which occurred in more than one patient [6]. Another study used transcriptome sequencing to identify a fusion gene, *ESRRA-C11orf20*, that occurs between neighboring genes and was shown to be present in 15% of patients with HG-SC [7]. Yet, it is unknown whether this fusion gene translates into a fusion protein or is cancer-specific as its presence/absence in non-cancerous tissues was not reported.

In this study, we adopted a strategy combining high-throughput paired-end transcriptome sequencing and stringent bioinformatic filtering to identify six novel fusion genes in HG-SC. Importantly, one of the fusion genes, *CDKN2D-WDFY2*, is present among 20% of 60 cancer samples analyzed and absent in non-cancer samples. This fusion gene is also expressed in OV-90, an established HG-SC cell line. A genomic breakpoint was identified in intron 1 of CDKN2D and intron 2 of WDFY2 in HG-SC patient sample, providing direct evidence that this is a fusion gene. Transfection of this fusion transcript leads to the loss of wildtype CDKN2D and wildtype WDFY2 protein expression, and a gain of a short WDFY2 protein isoform which appears to change the protein levels of PI3K/AKT pathway members. This is by far the most frequent HG-SC-specific fusion event that may have implications in a major signaling pathway that is known to be important for oncogenesis. The *CDKN2D-WDFY2* fusion gene could represent a molecular signature important for defining a major sub-lineage of HG-SC and may provide crucial insight into the underlying mechanism of this deadly disease.

## Results

### Identification of novel fusion transcripts in high-grade serous ovarian cancer

To identify fusion transcripts that are transcribed from fusion genes, we sequenced the transcriptome of seven cancer samples from patients with HG-SC. The cancer samples are primary tumors from patients that did not receive neoadjuvant chemotherapy prior to removal of diseased ovary. Since there is still debate about the cell of origin of HG-SC as histologically similar cancers have been identified on the surface of the ovary and fallopian tube [8], [9], we performed transcriptome sequencing using two control pools: one pool of RNA from ovaries of 20 non-cancerous donors and another pool of RNA from the fallopian tubes of 6 non-cancerous donors. We used the Illumina Genome Analyzer II for sequencing these samples and generated output sequences of paired 75 or 100 nucleotide sequence reads. In all, 9 lanes of Illumina Genome Analyzer yielded approximately 476 million reads that were uniquely mappable to the human genome (Table S1).

Our strategy for identifying fusion transcripts was to search for paired ‘chimeric’ reads with each read mapping to a different gene either in the genome or transcriptome. To minimize the cases of false positives, we used the following filters. First, an event was considered a fusion transcript only if it was supported by at least two paired chimeric reads. To avoid clonal duplicate sequence reads leading to spurious fusion calls, we required that the starting genomic positions of the corresponding paired reads be at least 5 base pairs apart. Second, two genes in theory can fuse in four different strand combinations; we required that the chimeric reads predominantly support only one of the four strand combinations of gene fusion. Third, we filtered out cases of overlapping genes and homologous genes with shared sequences. Lastly, we remapped the fusion supporting reads with BLAT [10] and ensured that the mappings could not find better or equally scoring mappings. This strategy led to the identification of nearly 356 putative fusion transcripts from the 7 cancer samples. Interestingly, the *ESRRA-C11orf20* fusion transcript, which is reported to be present in 15% of HG-SC patients, was absent in the 7 cancer samples sequenced. This could be due to either the small sample size or low level of expression of this fusion transcript such that it could not be detected in our samples. Importantly, our strategy was able to rediscover 16 previously identified fusion transcripts in ovarian cancer such as *LAMC2-NMNAT2* and *MAG-CD22* [6], indicating that the method is effective. The remainder of our identified 340 putative fusion transcripts have not been described before.

We selected 47 candidates out of 356 putative fusion transcripts for experimental validation based on the following criteria: 1) the fusion transcript should be present in at least two human cancer samples or supported by 3 or more chimeric reads, or 2) the fusion transcript includes one or more genes that are listed in the cancer gene catalog compiled by Memorial Sloan Kettering Cancer Center that implies functional association with cancer.

To validate the presence of these candidate fusion transcripts, we designed specific primers with each primer targeting one parental gene, therefore specifically amplifying the fusion transcripts but not the parental gene transcripts. We then employed RT-PCR to validate the presence of fusion candidates in the patient samples that were used for paired-end sequencing. For 15 candidates, we were able to obtain RT-PCR products indicating presence of the fusions at detectable levels. In most of these cases, we obtained a single RT-PCR band from the targeted fusion transcript, which was then excised and sequenced by Sanger sequencing. This led to the identification of the exact RNA junction of the fusion transcript (Table S2). The RNA junction sequences enabled us to search among the previously unmappable reads for paired ‘junction’ reads that could now specifically map to the junction site with one read, and to one of the fusion gene partners with another read. For most of the validated fusion transcripts, we were able to identify the corresponding junction reads, and this is shown in Table S3 along with the supporting paired chimeric reads. In all, 15 out of a total of 47 putative fusion transcripts chosen for validation were experimentally validated in patient samples used for sequencing. In addition to the HG-SC samples, we also sequenced two endometrioid and one clear cell cancer sample which are different subtypes of ovarian carcinoma. Analyses of the output sequences indicated that none of the 15 validated fusion transcripts found in HG-SC samples was present in endometrioid or clear cell cancer samples.

The known chromosomal instability of HG-SC is expected to lead to higher incidence of gene fusions. In the 15 validated fusion transcripts that we identified, 6 are inter-chromosomal recombinant events or long-distance intra-chromosomal recombinant events, suggesting that they are the result of major chromosomal rearrangements (Table 1). These were chosen for further study as they could represent true fusion genes. The remaining 9 validated fusion transcripts are neighboring gene chimeras. Previously we found that the majority of fusion transcripts resulting from neighboring genes have no evidence of local genomic rearrangement [11]. These candidates more likely result from transcriptional read-through or local aberrant trans-splicing, and therefore were not selected for current investigation.

**Table 1:** Validated fusion transcripts identified from transcriptome sequencing of HG-SC samples.

Fusion transcript	Type of fusion	Recurrence among 28 High-grade serous cancer samples	Recurrence among 10 non-cancer donor ovary samples	Recurrence among 4 non-cancer donor fallopian tube samples
*CDKN2D-WDFY2*	Interchromosomal (Chr 19/13)	12/60 (20%)*	0	0
*TMEM66-MSRB3*	Interchromosomal (Chr8/12)	5 (18%)	2	0
*FAM19A3-LPP*	Interchromosomal (Chr 1/3)	2 (7%)	0	0
*RFX2-CCDC94*	Intrachromosomal (Chr 19)	2 (7%)	0	1
*NR2F6-MAST3*	Intrachromosomal (Chr 19)	2 (7%)	1	0
*WDFY2-S1PR5*	Interchromosomal (Chr 13/19)	1 (3.5%)	0	0

Table shows the chromosomal locations, and frequency of occurrence for each fusion transcript in 28 HG-SC samples, 10 non-cancerous donor ovary samples, and 4 non-cancerous donor fallopian tube samples as determined by nested RT-PCR. *CDKN2D-WDFY2* indicated by “*” was validated in 60 instead of 28 HG-SC samples.

### *CDKN2D-WDFY2* occurs in multiple cancer samples

**Figure 1:**
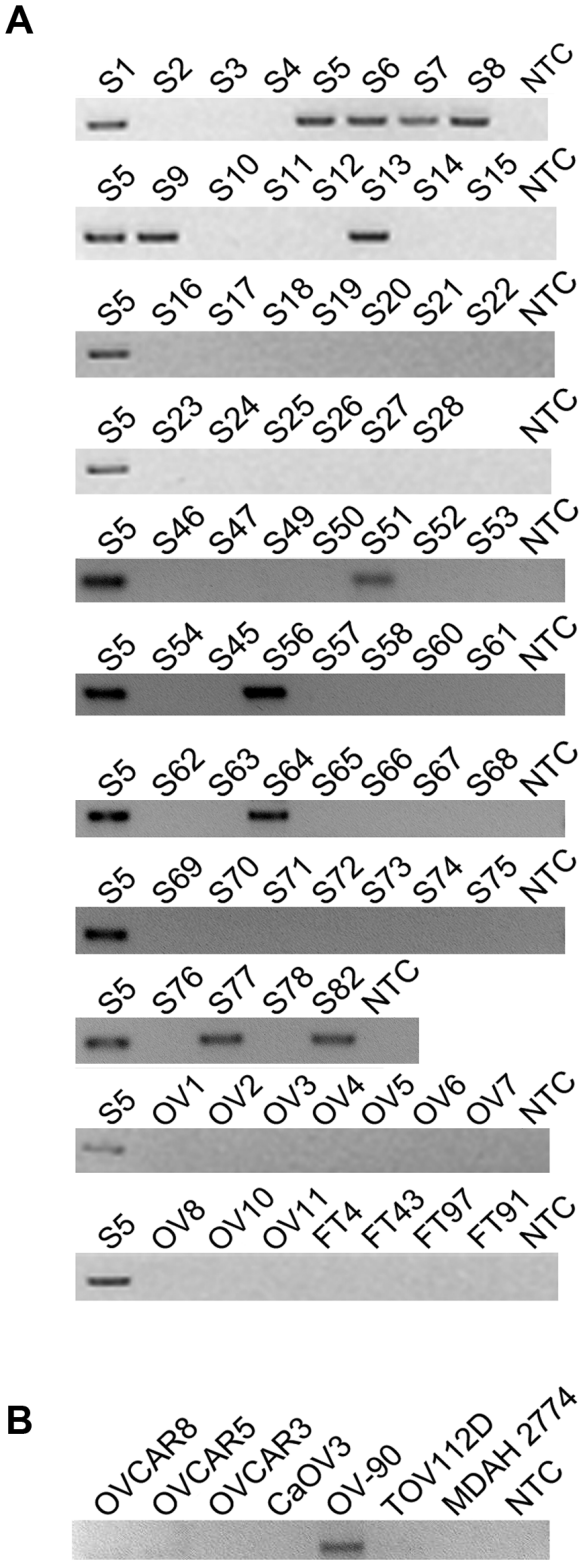
*CDKN2D-WDFY2* is a highly frequent fusion transcript in HG-SC cancer samples and cell line. (A) The results of nested RT-PCR for *CDKN2D-WDFY2* in 60 HG-SC samples (denoted by “S”), 10 non-cancerous donor ovary samples (“OV”), and 4 non-cancerous donor fallopian tube (“FT”) samples are shown. NTC refers to “no template control”. S5 is the sample in which the fusion transcript was initially identified and this serves as the positive control. (B) The results of RT-PCR for *CDKN2D-WDFY2* in five HG-SC cell lines (CaOV3, OV90, OVCAR8, OVCAR5 and OVCAR3), in addition to two endometrioid type cell lines (TOV112D and MDAH 2774) are shown.

To evaluate the frequency of occurrence of these 6 fusion gene candidates, we tested their expression in a cohort of 28 HG-SC patient samples by nested RT-PCR, which provides highly sensitive and specific amplification. In parallel, nested RT-PCR was also performed on a cohort of 10 non-cancerous donor ovary samples and 4 non-cancerous donor fallopian tube samples. Our results indicate that two of the six fusion transcripts were found only among the cancer cohort (Table 1) implying that they are cancer-specific. Among them, *CDKN2D-WDFY2* appeared to be a high incidence event. To further evaluate its frequency of occurrence, we increased the cohort size to 60 HG-SC samples and found that this fusion transcript was present in 20% (12 out of 60) of HG-SC samples and absent in all non-cancerous ovary and fallopian tube samples, suggesting that it is cancer-specific (Figure 1A). The remaining fusion transcripts displayed lower frequency of occurrence or non-cancerous specific pattern (Table 1). For example, *FAM19A3-LPP*, *RFX2-CCDC94* and *NR2F6-MAST3* were present in only 7% of the samples (Table 1 and Figure S1). *TMEM66-MSRB3* was present in 18% of the cancer samples, but it was also expressed in two of the non-cancerous ovary controls.

Because *CDKN2D-WDFY2* appeared to be a highly frequent event, we speculate that this fusion transcript may also be present in established HG-SC cell lines. This indeed is the case. By RT-PCR screening of five serous type cell lines (CaOV3, OV-90, OVCAR8, OVCAR5 and OVCAR3), in addition to two endometrioid type cell lines (TOV112D and MDAH 2774), we found that *CDKN2D-WDFY2* fusion transcript is expressed in OV-90, but not others (Figure 1B). The presence of *CDKN2D-WDFY2* in an established HG-SC cell line such as OV-90 may further support the potential significance of the *CDKN2D-WDFY2* fusion in HG-SC.

### *CDKN2D-WDFY2* is a fusion gene resulting from a chromosomal rearrangement

Since *CDKN2D-WDFY2* is an inter-chromosomal fusion event in HG-SC that appears to be cancer-specific and occurs at a frequency that is without precedent in ovarian cancer, we chose to examine it in detail. The identified RNA junction sequence using RT-PCR and Sanger sequencing indicates that exon 1 of CDKN2D is fused to exon 3 of WDFY2 mediated by splicing, and this junction is identical among all patients carrying this fusion transcript. To investigate whether this is the only RNA junction, we analyzed the transcriptome sequencing data from patient S5 where this fusion transcript was highly expressed and our analysis revealed no other RNA junction. In addition, RT-PCR performed on patient S5 using primer pairs targeting different exons of parental genes also revealed only the same RNA junction. Thus, the results suggest that there is only one RNA junction produced from *CDKN2D-WDFY2* fusion.

**Figure 2:**
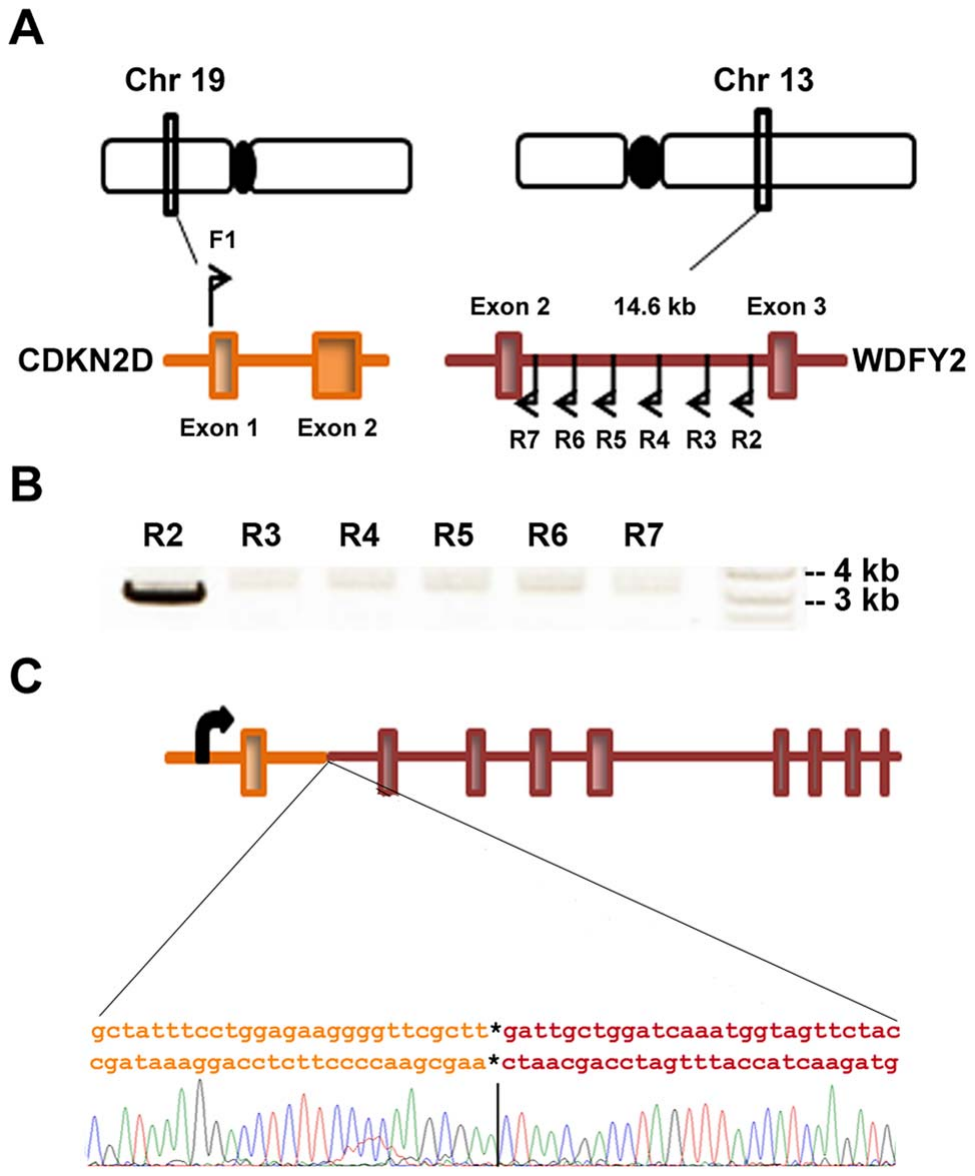
*CDKN2D-WDFY2* fusion transcript results from a genomic rearrangement of chromosome 19 and 13. (A) *CDKN2D* gene is present on chromosome 19 and contains two exons while *WDFY2* is present on chromosome 13 and contains 12 exons. The RNA junction indicates a fusion between exon 1 of CDKN2D and exon 3 of WDFY2. To identify the genomic breakpoint, a forward primer F1 was designed in exon 1 of CDKN2D and several reverse primers were designed in the intron between exon 2 and 3 of WDFY2. (B) Results from long range PCR on genomic DNA from patient S5 are shown using primer F1 paired with different reverse primers. A product is seen when R2 is used as the reverse primer. (C) Schematic of *CDKN2D-WDFY2* genomic breakpoint with the junction sequence and trace identified by Sanger sequencing of the product in (B).

*CDKN2D* is located in chromosome 19 whereas *WDFY2* in chromosome 13. To establish whether this fusion transcript indeed results from chromosomal rearrangement and not from trans-splicing, we searched for the genomic breakpoint of *CDKN2D-WDFY2* in the tumor from patient S5. As illustrated in Figure 2A, several primers were designed to target different locations in intron 2 of WDFY2 (∼14 kb long), and these primers were paired with a common primer targeting exon 1 of CDKN2D (CDKN2D intron is comparatively short at ∼1 kb). As shown in Figure 2B, long-range PCR performed using one particular primer combination on patient tumor genomic DNA led to a single amplified band of approximately 3 kb. Gel purification and Sanger sequencing of this band revealed the precise genomic breakpoint which is located in the intron 679 bp from the end of exon 1 of CDKN2D (chr19:10,678,510, hg19 version) and in the intron 3931 bp upstream from the exon 3 of WDFY2 (chr13:52,245,375, hg19 version) (Figure 2C and Table S2). This single genomic breakpoint observed in patient S5 provided the direct evidence that *CDKN2D-WDFY2* is a fusion gene resulting from a near clonal chromosomal rearrangement. Further sequence analysis of the breakpoint revealed no obvious sequence homology or microhomology around the breakpoint, suggesting that the recombination is possibly mediated by non-homologous end joining (NHEJ) [12]. To answer the question whether the same identified genomic breakpoint occurs in other HG-SC patient samples, we analyzed genomic DNA from 5 patient samples that express the fusion transcript using primers specific for this identified genomic breakpoint. However, none of these samples produced the expected PCR band or unexpected bands. This indicates that locations of genomic breakpoints likely vary among cancer samples and would require different specific primer pairs to probe their locations. This is similar to what was found in prostate cancer in which the genomic breakpoints of *TMPRSS2-ERG* fusion gene was shown to differ among 29 patient samples, with none of them occurring at the same location [13].

### *CDKN2D-WDFY2* led to the loss of CDKN2D protein expression and a gain of a shortened WDFY2 protein isoform

**Figure 3:**
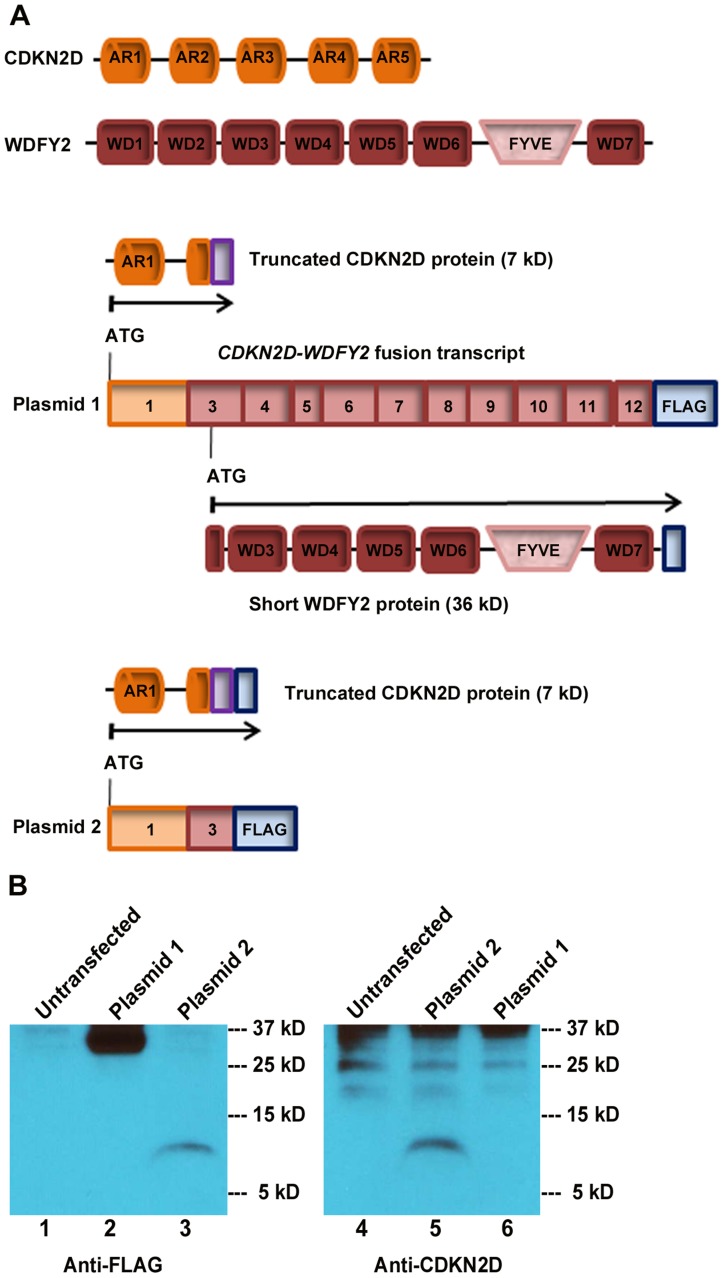
*CDKN2D-WDFY2* fusion transcript gives rise to a short WDFY2 protein isoform. (A) Protein domain structure of CDKN2D and WDFY2. CDKN2D consists of five Ankyrin repeats (AR1-5). WDFY2 contains seven WD-repeats (WD1-7) that are involved in protein-protein interactions and a FYVE domain for binding to phosphatidylinositol-3-phosphate on vesicular membranes. Potential translational consequences of *CDKN2D-WDFY2* fusion transcript (plasmid 1) include a truncated CDKN2D protein (7 kD) that starts from the original ATG in CDKN2D ORF (orange) and is fused to an out-of frame exon of WDFY2 (purple), and a short WDFY2 protein (36 kD) that is translated in the original frame starting from an internal cryptic ATG in exon 3 of WDFY2. Plasmid 2 which contains only the ORF for the truncated CDKN2D protein is used as a control. (B) Protein assay of untransfected HEK-293T cells (lane 1 and 4), plasmid 1 transfected (lane 2 and 6), and plasmid 2 transfected cells (lane 3 and 5) with the indicated antibodies. Plasmid 1 transfection led to a 36 kDa protein indicating that short WDFY2 protein isoform is translated (lane 2), while the predicted truncated CDKN2D fusion (7 kD) is not selected for translation (lane 6). Truncated CDKN2D fusion protein is detected only when the expression of plasmid 2 was visualized by anti-FLAG (lane 3) or a commercial anti-CDKN2D antibody (lane 5). Endogenous CDKN2D has a size of 19 kD. Bands at 25 kD and above in lanes 4 to 6 are non-specific bands.

The high rate of occurrence of *CDKN2D-WDFY2* among patient tumors suggests that this gene fusion could play a role in oncogenesis. An important yet unanswered question is whether this gene fusion leads to the translation of an aberrant protein. The fusion of exon 1 of CDKN2D to exon 3 of WDFY2 could have two major translational consequences based on the analysis of ‘start’ and ‘stop’ codon in the fusion transcript. The first is a protein of a truncated CDKN2D with the addition of 16 new amino acids from the subsequent out-of-frame WDFY2 sequence, giving rise to a 7 kD protein (Figure 3A). The second is a short WDFY2 protein resulting from an internal translational initiation site that is in frame with the parental gene stop codon (Figure 3A). To investigate the translational consequences of the fusion gene, we cloned the full-length fusion transcript (from patient S5) that encompasses both ORFs under the control of CMV promoter (plasmid 1). A FLAG tag was inserted at the C-terminus in frame with WDFY2. A second plasmid was constructed under the control of the same CMV promoter but contained only the ORF for the truncated CDKN2D with a C-terminal FLAG tag (plasmid 2). The truncated CDKN2D protein expressed from plasmid 2 can be detected by both anti-FLAG antibody and a commercial anti-CDKN2D antibody, and the same truncated protein expressed from plasmid 1 by anti-CDKN2D antibody. The short WDFY2 protein expressed from plasmid 1 was visualized by anti-FLAG antibody. Transfection of plasmid 1 and the subsequent Western analysis using anti-FLAG antibody revealed a 36 kDa protein (Figure 3B, lane 2) that is absent in the untransfected cells (Figure 3B, lane 1), indicating that this short WDFY2 protein is indeed translated and has the predicted size. In contrast, anti-CDKN2D antibody revealed that the truncated CDKN2D ORF is not selected for translation when plasmid 1 is transfected (Figure 3B, lane 6), even though it is the first ORF encountered by translational machinery in the fusion transcript. This is not due to antibody recognition issues as the commercial antibody used can readily recognize the truncated CDKN2D protein when plasmid 2 containing only this ORF is transfected (Figure 3B, lane 3 and 5). Consistent with the observation in transfected cells, protein analysis of tissue from patient S5 also failed to reveal the presence of this 7 kD predicted truncated CDKN2D protein (Figure S2, compare lane 1 to lane 3). However, our efforts to identify the short WDFY2 protein in patient S5 were inconclusive. We tested three commercially available anti-WDFY2 antibodies that potentially could recognize not only full-length WDFY2 but also short WDFY2. Yet our western blot results on HEK-293T cells overexpressing short WDFY2 (plasmid 1) showed that these antibodies failed to recognize the short isoform (Figure S3). Due to this limitation, we are unable to analyze the presence/absence of endogenous short WDFY2 protein isoform in patient S5 although the corresponding fusion transcript is clearly present.

**Figure 4:**
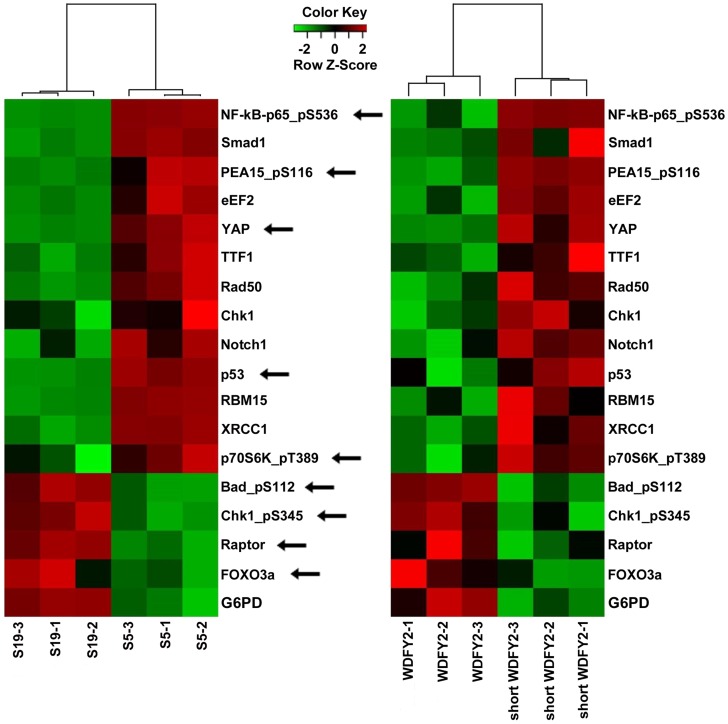
Proteins that are altered consistently in patient tissues and transfected cell lines by short WDFY2. Heatmaps show the results of a set of 17 proteins identified by RPPA analysis from a total of 130 proteins. These proteins are significantly altered when comparing patient tissue S5 (expressing *CDKN2D-WDFY2* fusion transcript) to S19 (does not express fusion transcript), as well as OVCAR8 cell line transfected with short WDFY2 to that transfected with wildtype WDFY2. The antibodies used in this analysis are indicated on the right. Green and red indicate lower and higher expression on a relative scale. The experiment was performed in triplicates. Arrows point to members of PI3K/AKT pathway which are highly represented in this set of 17 proteins (hypergeometric test p-value = 0.0205).

WDFY2 is an endosomal protein with seven WD repeats that is thought to function as a ‘docking station’ that facilitates the interactions between kinases and their substrates. In particular, studies have shown that WDFY2 can bind to AKT and its substrates [14], [15]. We hypothesized that the short WDFY2 protein isoform, which contains only five of the seven WD repeats, may affect the interaction of AKT with its substrates and thus alter downstream signaling. To probe the functional differences of short WDFY2 versus wildtype WDFY2 on signaling pathways, we performed reverse phase protein arrays (RPPA) [16], which provides a means to quantitatively assess the levels of 130 cancer-associated proteins using 163 distinct antibodies. The assay was performed on a HG-SC cell line (OVCAR8) transfected with short WDFY2 as compared to wildtype WDFY2, and patient samples S5 (expressing *CDKN2D-WDFY2* fusion transcript) as compared to S19 (not expressing the fusion transcript). The analysis revealed 99 proteins that were significantly changed between patients S19 and S5, and 53 proteins between cell lines transfected with wildtype WDFY2 and short WDFY2. To identify those among the significantly changed proteins that could result from the expression of short WDFY2, we searched for those proteins that are altered in the same manner in both transfected cell lines and patient samples. This led to a set of 17 proteins whose differential expression levels are shown in Figure 4. As controls, the expression level of three of these proteins were further confirmed by traditional western analysis (Figure S4). In addition, we confirmed that OVCAR8 cells subjected to RPPA analysis have similar levels of transfected wildtype and short WDFY2 (Figure S5). Therefore, the difference found in RPPA analysis is not due to different protein expression level of wildtype and short WDFY2 in cell line, but likely attributed to their difference in protein activity. However, we are unable to confirm the levels of endogenous wildtype and short WDFY2 protein isoform in patient S5 and S19 due to the unavailability of suitable antibodies.

To investigate which signaling pathways might be significantly altered by the expression of short WDFY2, we performed pathway analysis on the RPPA data. The result indicates that members of the PI3K/AKT pathway are highly represented in this set of 17 proteins that shows the same alteration in transfected cell lines and patient tissues (hypergeometric test p-value = 0.0205). For instance, BAD and FOXO3A, both substrates of AKT, were significantly changed in cell line expressing short versus wildtype WDFY2 and in patient S5 versus S19. This result indicates that the expression of short WDFY2 may alter the PI3K/AKT pathway which may in turn contribute to tumor progression in HG-SC.

## Discussion

High-grade serous cancer is characterized by a high degree of heterogeneity among tumors and massive genome rearrangements. This could be related to *TP53* as mutations in this gene, seen in almost 96% of high-grade serous tumors, are known to associate with genomic instability. Mutations in common cancer genes such as *PTEN*, *BRCA1*, *BRCA2*, are also present but at much lower prevalence in HG-SC. However, recurrent mutations specific only to HG-SC but not other cancers have been difficult to identify, presumably due to the high heterogeneity among tumors. In contrast, cancers such as chronic myeloid leukemia and prostate cancer can be stratified by a cancer type-specific fusion such as *BCR-ABL* and *TMPRSS2-ERG* respectively [3]. Prior to this study, only one fusion gene, *ESRRA-C11orf20*, has been found to occur at 15% in HG-SC [7]. This fusion involves two neighboring genes. However, our analysis showed that this fusion transcript was absent in our sequenced cancer samples presumably due to its lower frequency of occurrence. Moreover, the significance of this fusion for cancer progression is uncertain because it is yet to be established whether it is cancer-specific and translates into a protein product, or present in an established high-grade serous type cell line. The *CDKN2D-WDFY2* fusion gene, that we identified by RNA sequencing and validated experimentally in a cohort of 60 patient samples, represents the most frequent cancer-type specific mutation for high-grade serous cancer. Three key features are associated with this recombinant event. First, it is recurrent among 20% of all HG-SC tumors, a significant frequency given the highly heterogeneous nature of this disease. Second, the exact same RNA junction is observed in the fusion transcript across patients suggesting that this mutation leads to a specific aberrant protein function. Third, it is not present in the non-cancerous ovaries or fallopian tubes. All of these features suggest that this gene fusion, alone or in combination with other mutations, could play a role in cancer progression, perhaps by providing survival advantage to cancer cells.

Our experiments show that the *CDKN2D-WDFY2* fusion leads to the loss of translation of wild type CDKN2D and wild type WDFY2, and a gain of a short WDFY2 protein isoform presumably under the control of the CDKN2D promoter. Loss of CDKN2D function can affect both cell cycle regulation as well as DNA repair. CDKN2D (cyclin-dependent kinase inhibitor 2D, or p19 or INK4D) is known to regulate cell-cycle by competing with D-type cyclins for binding to CDK4/6 and to regulate the G1/S transition [17]. CDKN2D also has a distinct role in DNA repair, as its levels are upregulated during genotoxic stress, and the high levels are required for efficient DNA repair [18], [19]. The loss of functional CDKN2D would mean diminished ability to repair DNA damage that could lead to increased gene mutations and chromosomal recombinations in HG-SC. However, CDKN2D null mice do not develop spontaneous tumors [20]. This indicates that loss of CDKN2D may need to be combined with other common mutations, such as p53 seen in 96% of high-grade serous tumors, to result in a high degree of DNA damage/genome instability that is the hallmark of HG-SC.

WDFY2 contains seven WD (tryptophan-aspartic acid dipeptide) repeats that are thought to form a circularized beta propeller structure. In addition, it also contains a FYVE domain that binds to PI3P (phosphatidylinositol 3-phosphate) on endosomal membranes [21]. The WD repeats have been shown to serve as a docking platform for the interaction of AKT and its substrates [14], [15]. Our RPPA data on patient samples and transfected cell lines, which showed that the gain of a short WDFY2 protein may alter the PI3K/AKT pathway, seems to support the above association of WDFY2 with AKT. However, the current study is not able to establish the presence of short WDFY2 protein in patient samples due to the lack of useful antibodies. Future work would require establishing this missing link in this hypothesis.

The observation that the *CDKN2D-WDFY2* fusion transcript exhibited the same RNA junction in patients carrying this fusion transcript and the absence of any other splice variant suggests that this fusion transcript needs to be made precisely, that is, connecting exon 1 of CDKN2D and exon 3 of WDFY2. Our results show that this specific connection, which eliminates the original start codon of WDFY2 in the transcript, may lead to the gain of translation of a short WDFY2 protein isoform. Furthermore, the short isoform is presumably under the control of the CDKN2D promoter, thus its expression could be tightly tuned to cell-cycle. Both the short isoform and the misregulation by a cell-cycle dependent promoter could result in an aberrant WDFY2 function affecting PI3K/AKT pathway. Thus, the loss of wildtype CDKN2D and wildtype WDFY2 in combination with the gain of a misregulated short WDFY2 isoform would explain why the fusion gene occurs in 20% of HG-SC tumors, a significant frequency considering the highly heterogeneous nature of HG-SC. A clear cancer phenotype may manifest itself only when the combined context of p53 mutation, loss of CDKN2D, loss of wildtype WDFY2, and gain of misregulated short WDFY2 are present together.

This fusion gene has several potential clinical utilities. It could be used in stratification of this disease, i.e. in identifying subtypes of HG-SC patients, thus allowing personalized treatment using tailored therapeutics. If proven to be oncogenic, this short WDFY2 protein could also serve as a therapeutic target for small molecule drugs. Lastly, *CDKN2D-WDFY2* could be used as a clinical biomarker for detection of a substantial fraction of HG-SC, as this specific molecular signature might be present in circulating cancer cells or in local body fluids released from tumor mass thus detectable using non-invasive assays. A specific molecular signature for detection of ovarian cancer would have major clinical implications given that much of mortality in ovarian cancer is due to its late detection.

## Materials and Methods

### Ethics statement

All tumor samples and non-cancer samples were collected following procedures approved by IRB at Baylor College of Medicine.

### Human high-grade serous ovarian cancer samples and cell lines

Anonymized ovarian cancer tissue samples were obtained from the Tissue Acquisition and Distribution Core of the Dan L. Duncan Cancer Center and Department of Pathology and Immunology and the Gynecologic Oncology Group under an approved Baylor College of Medicine Institutional Review Board protocol. The patient tissues are all fresh frozen samples. All tumor samples were confirmed to have greater than 80% serous adenocarcinoma prior to processing. RNA was extracted from cancer samples and non-cancerous donor samples using Ribopure kit (Ambion).

OVCAR8 cell line was maintained in RPMI-1640 supplemented with 10% FBS and 1% Penicillin/Streptomycin. HEK-293T was maintained in DMEM supplemented with 10% FBS and 1% Penicillin/Streptomycin.

### RNA processing for paired-end transcriptome sequencing

Total RNA samples with RNA integrity number (RIN) of 8 and higher were used for transcriptome sequencing using Illumina mRNA-seq protocol. Briefly, 5 µg of total RNA was used to isolate mRNA using Sera-mag Magnetic Oligo(dT) beads. mRNA was then fragmented and converted into double-stranded cDNA. Adapters were ligated to the double-stranded cDNA and this library was then size-selected to obtain fragments in the range of 200–500 bp. Finally, PCR amplification was performed to obtain the final cDNA library. 10 nM of the library was then used for sequencing. Sequencing of samples S3, S4, S5, S6, CC2, EC2 and EC4 was performed on the Illumina genome analyzer II (GAII) at the Center for Cancer Epigenetics Solexa Sequencing Core located in the University of Texas- M.D. Anderson Cancer Center with an output of paired end 75-nucleotide reads. Sequencing of the rest of the samples was performed at the Genomic and RNA Profiling Core at Baylor College of Medicine with an output of paired end 100 nucleotide reads.

### Bioinformatic identification of gene fusions

RNA-Seq reads were processed by employing the following filters in order: 1) trimming by base quality score in 5′ to 3′ direction, using 15 as minimum threshold, 2) removing reads smaller than 45 basepairs. We obtained roughly 476 million reads uniquely mappable to the human genome UCSC hg19/NCBI chr37 (Table S1). Reads were first mapped to the transcriptome using Pash 3.0 [22]. Reads pairs mapping to non-overlapping genes with 0 mismatches were preserved as inconsistent reads; reads mapping to the same gene or overlapping genes were discarded from analysis. Reads with at most one end mapping to a gene were further selected, and mapped to the genome using bwa [23]. Again reads mapping to the same gene or overlapping genes were discarded, whereas reads mapping to two different genes were selected. Inconsistent read pairs derived from either transcriptome or genomic mapping were then combined, and non-overlapping gene pairs with at least two read pairs spanning them were selected as candidate gene fusions. We then used the filters (described in the results section) to reduce false positives and thus identified the 356 putative fusion transcripts from the 7 serous cancer samples.

### Identification of junction reads

RNA junctions were accurately defined using RT-PCR and Sanger sequencing, and then used as templates to align junction reads. Reads that were earlier unmappable to the genome and transcriptome were aligned to the PCR amplicon. A paired read was considered as a junction read only if it met the following conditions: 1) one read of the paired read mapped to either parental gene of the chimeric RNA. 2) Junction read should overlap with at least six nucleotides of the sequence on either side of the RNA junction. 3) Mismatch tolerance was set at two mismatches, but for the six nucleotides flanking the RNA junction, no mismatches were tolerated.

### RT-PCR

1 µg of RNA was used for each reverse transcription reaction. RNA was incubated with Oligo dT and dNTPs and denatured at 65°C. This was followed by the addition of a master-mix containing 1× superscript buffer, 10 mM DTT, 5 mM Magnesium chloride, RNaseOUT and Superscript III reverse transcriptase. Reactions were then incubated at 50°C for 50 minutes. Reactions were terminated by incubation at 85°C for 5 minutes. cDNA was then treated with RNase-H for 20 minutes at 37°C. 1 µl of cDNA was used as template for PCR using the primers listed in Table S4. PCR master mixes included 3% DMSO and PCR was done using a standard three-step protocol with annealing temperature of 56°C. The products of RT-PCR were gel purified and sequenced by Sanger sequencing to identify the exact fusion junctions of the candidate events. A “no template” reaction was also conducted and used as negative control.

### Long range PCR

Using the primers listed in Table S4, we performed long-range PCR on 200 ng of genomic DNA. LA PCR kit (Takara) was used for these reactions and reactions were performed according to the manufacturer's protocols. Two-step PCR was performed with annealing and extension at 68°C for 20 minutes. Products were run on gels and then gel purified and sequenced by Sanger sequencing.

### Cloning and transfection

Constructs were made for truncated CDKN2D, CDKN2D-WDFY2, wildtype WDFY2 and short WDFY2. Using the primers listed in Table S4, RT-PCR was performed on patient S5 RNA to generate the products. A C-terminal FLAG tag was added to all PCR products and they were cloned into the vector HDM-luc [24] which has a CMV promoter. HEK-293T cells were transfected with the indicated plasmids using TransIT- 293 reagent (Mirus) according to the manufacturer's protocol. For RPPA experiments, OVCAR8 cell line was transfected with either WDFY2-FLAG or short WDFY2-FLAG using the Fugene 6 transfection reagent (Roche).

### Protein extraction and western blotting

48 hours after transfection, proteins were extracted using RIPA buffer (Santa cruz biotechnology). Briefly, cells were washed with PBS and then RIPA buffer (supplemented with Sodium vanadate, PMSF and protease inhibitors) was added to cells and lysis was allowed to continue for 5 minutes on ice. Then, cells were scraped and collected in Eppendorf tubes and centrifuged at 8000× g for 10 minutes at 4°C. The supernatant was collected and used in western blotting.

For western blotting, equal amounts of lysates were run on a 4–20% Tris-glycine gel (Bio-rad). Proteins were transferred onto nitrocellulose membrane using CAPS buffer (VWR) at 100 V for 1 hour. Membrane was rinsed with 1× TBS and then blocked with 1× TBST containing 5% nonfat dry milk for 2 hours. This was followed by three washes with 1× TBST. Primary antibodies in blocking buffer were incubated with the membrane overnight at 4°C. Following three washes with 1× TBS/T, membrane was incubated with secondary antibodies in blocking buffer for 2 hours. Finally after three washes, detection reagents (Supersignal West Femto from Thermo Scientific) were incubated with the membrane and then exposed to film.

The following antibodies were used: Anti-FLAG (SIGMA F1804), Anti-CDKN2D (Abcam ab102842), Anti-WDFY2 (P-17 (sc-84659) and C-20 (sc-84658) - both from Santa cruz biotechnology), Anti-WDFY2 (Center from Abgent #AP5783c), Anti-rabbit IgG-HRP (Cell Signaling #7074) and Anti-mouse IgG-HRP (Cell Signaling #7076).

### Reverse phase protein array (RPPA)

Reverse phase protein array (RPPA) experiment was performed at the University of Texas MD Anderson Cancer Center RPPA core using the antibodies listed in Table S5. Tumor or cell lysates (assayed in triplicate) were two-fold-serial diluted for 5 dilutions (from undiluted to 1∶16 dilution). Serial diluted lysates were arrayed on nitrocellulose-coated slides and each slide was probed with a validated primary antibody plus a biotin-conjugated secondary antibody. Only antibodies with a Pearson correlation coefficient between RPPA and western blotting of greater than 0.7 were used in RPPA. The signal obtained was amplified using a Dako Cytomation–catalyzed system and visualized by DAB colorimetric reaction. The slides were scanned, analyzed, and quantified using a customized-software Microvigene to generate spot intensity. Relative protein levels for each sample were determined by interpolation of each dilution curves from the standard curve by utilizing the R software package Supercurve [25], [26]. All the data points were further normalized for protein loading. We determined antibodies with significant changes between the tested conditions by employing the Mann-Whitney-Wilcoxon test (p<0.05), using the R statistical system. We further identified antibodies that are significantly changed (p<0.05) and in the same direction between the patient samples S5 and S19 and the cell line samples transfected with either full-length WDFY2 or short WDFY2. Pathway analysis was based on the NCI pathway interaction database (http://pid.nci.nih.gov/search/pathway_landing.shtml?what=graphic&jpg=on&pathway_id=pi3kciaktpathway) and Cell Signaling AKT substrate database (http://www.cellsignal.com/reference/pathway/akt_substrates.html). The enrichment of the PI3K/AKT pathway among the identified 17 protein set was determined using the hypergeometric test. RPPA validation was performed on protein extracts from patient tumors S5 and S19 as well as OVCAR8 overexpressing either wildtype WDFY2 or short WDFY2 using the following antibodies: PEA15_pS116 (Invitrogen #44-836G), RBM15 (Novus Biologicals #21390002) and NF-kBp65_pS536 (Cell Signaling #3033).

## Supporting Information

Figure S1 Recurrence of the 5 validated fusion transcripts in HG-SC. Figure shows the results of nested RT-PCR for the indicated fusion transcripts in 28 high-grade serous cancer samples (denoted by “S”), 10 non-cancerous donor ovary samples (“OV”) and 4 non-cancerous donor fallopian tube (“FT”) samples. NTC refers to “no template control”. In the case of *TMEM66-MSRB3*, several non-cancerous samples displayed PCR bands. However, only OV1 and OV2 (designated by asterisk) contained the true fusion transcript as confirmed by Sanger sequencing of the bands.
(DOCX)

Figure S2 Truncated CDKN2D protein was not observed in patient sample. Truncated CDKN2D protein can be visualized by a commercial anti-CDKN2D antibody and anti-FLAG antibody when plasmid 2 is transfected. However, band expected from truncated CDKN2D protein is not observed in protein extracts from patient S5.
(DOCX)

Figure S3 Commercial antibodies to WDFY2 do not recognize short WDFY2 protein isoform produced from the fusion transcript. Protein assay of untransfected HEK-293T cells and plasmid 1 transfected HEK-293T cells with the indicated antibodies. All three WDFY2 antibodies did not recognize the short WDFY2 protein isoform (as seen in the FLAG western) but recognized a number of non-specific bands leading to the conclusion that these antibodies could not be used on patient tissues.
(DOCX)

Figure S4 Validation of RPPA results. Western blots were performed using the indicated antibodies to confirm the results from RPPA study. Results confirmed that these three proteins are upregulated in the patient sample S5 (containing CDKN2D-WDFY2 fusion gene) as compared to sample S19 (which does not contain the fusion gene). Similarly, these proteins are upregulated in OVCAR8 transfected with short versus wildtype WDFY2. The differences between the tissue samples are more pronounced than the cell line samples, and this is consistent with the quantitative data obtained by RPPA.
(DOCX)

Figure S5 Controls for RPPA experiment. Western blot using FLAG antibody on OVCAR8 cells transfected with either wildtype WDFY2 or short WDFY2. Similar levels of transfected protein are observed in both cases.
(DOCX)

Table S1 Statistics of output reads from high-throughput transcriptome sequencing. The raw reads, usable reads, and the uniquely mapped reads that met our stringent criteria are shown, obtained for each sample by paired-end sequencing. S refers to HG-SC sample while CC refers to the clear-cell ovarian cancer sample and EC refers to endometrioid ovarian cancer sample.
(DOCX)

Table S2 Junction sequences of validated fusion transcripts in HG-SC samples. RNA junction sequences as determined by RT-PCR and Sanger sequencing are shown for each fusion transcript. The junction is denoted by an asterisk. For *CDKN2D-WDFY2*, the sequence of the genomic breakpoint is also shown by an asterisk.
(DOCX)

Table S3 Paired chimeric reads and junction reads supporting fusion transcripts from each patient sample. The number of paired chimeric reads and junction reads obtained for each of the 15 fusion transcripts contributed by each sequenced sample is shown. For example, “7+19” indicates 7 paired chimeric reads and nineteen junction reads for the fusion transcript *FAM19A3-LPP* in sample S3.
(DOCX)

Table S4 Primers used in experiments.
(DOCX)

Table S5 Antibodies used in RPPA experiments.
(DOCX)
