# Supplementary figures and images for: CDKN2D-WDFY2 Is a Cancer-Specific Fusion Gene Recurrent in High-Grade Serous Ovarian Carcinoma
Source: PLoS Genet. 2014 Mar 27;10(3):e1004216. doi: 10.1371/journal.pgen.1004216 (PMC3967933; doi:10.1371/journal.pgen.1004216)

**Figure S1**

**
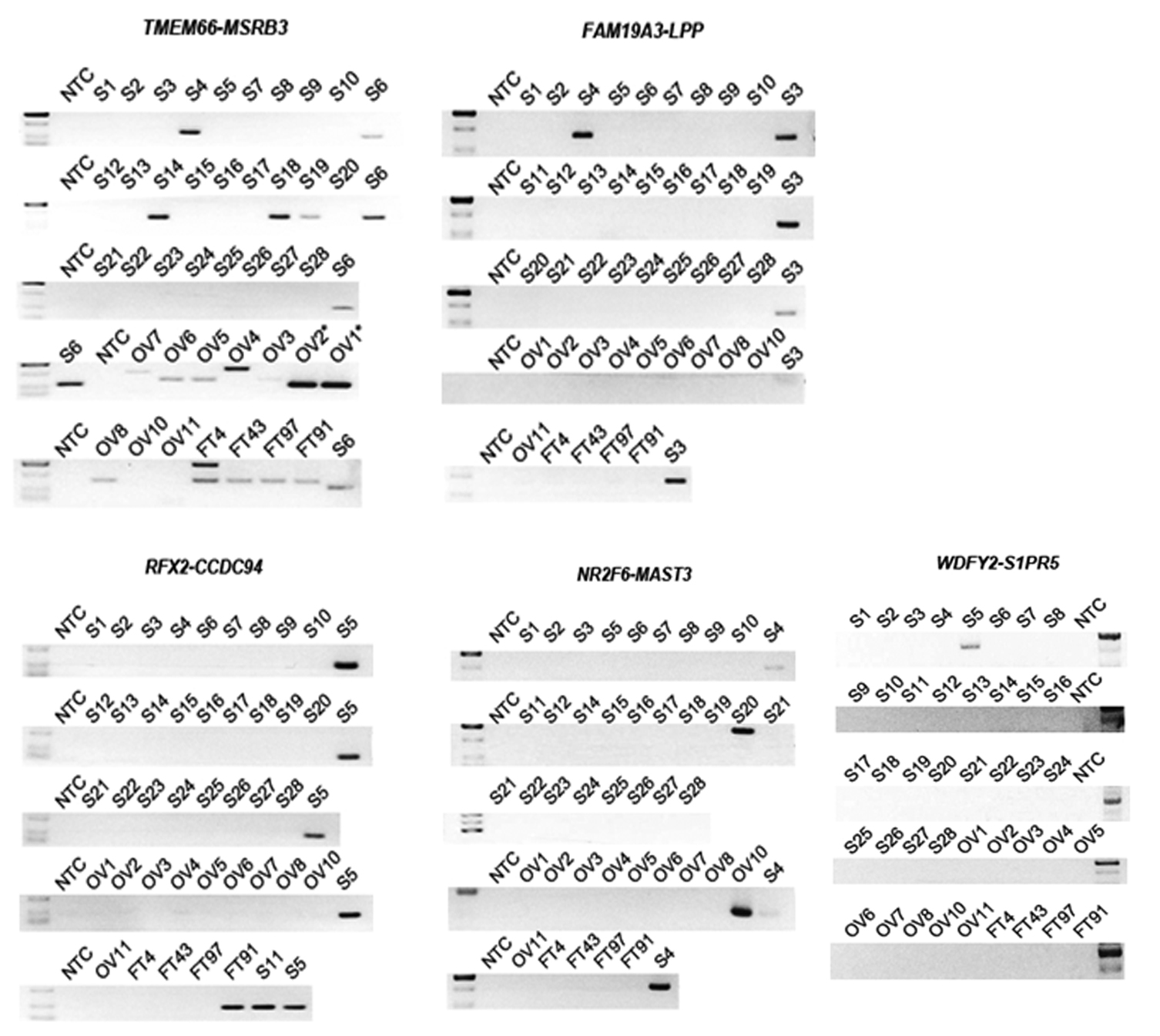
**

Supplement: Figure S1 — Recurrence of the 5 validated fusion transcripts in HG-SC. Figure shows the results of nested RT-PCR for the indicated fusion transcripts in 28 high-grade serous cancer samples (denoted by “S”), 10 non-cancerous donor ovary samples (“OV”) and 4 non-cancerous donor fallopian tube (“FT”) samples. NTC refers to “no template control”. In the case of TMEM66-MSRB3, several non-cancerous samples displayed PCR bands. However, only OV1 and OV2 (designated by asterisk) contained the true fusion transcript as confirmed by Sanger sequencing of the bands. (DOCX) [file pgen.1004216.s001.docx]

**Figure S2**


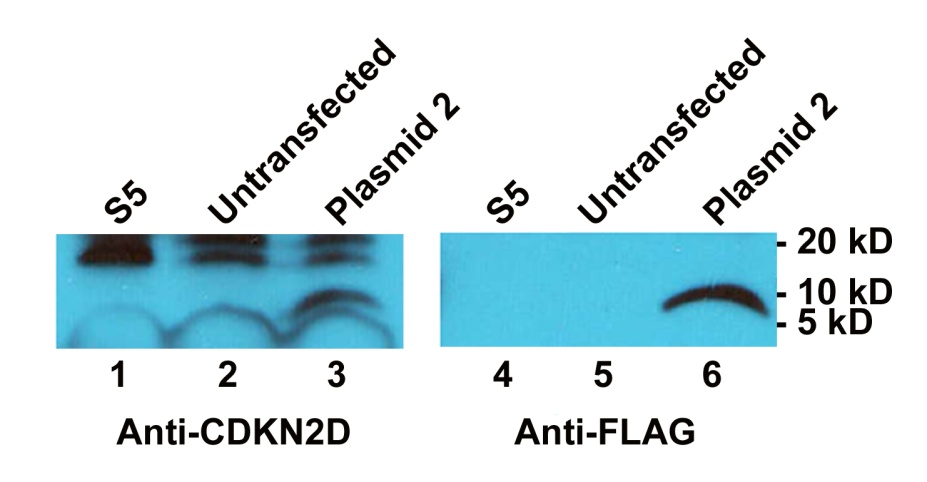

Supplement: Figure S2 — Truncated CDKN2D protein was not observed in patient sample. Truncated CDKN2D protein can be visualized by a commercial anti-CDKN2D antibody and anti-FLAG antibody when plasmid 2 is transfected. However, band expected from truncated CDKN2D protein is not observed in protein extracts from patient S5. (DOCX) [file pgen.1004216.s002.docx]

**Figure S3**

**
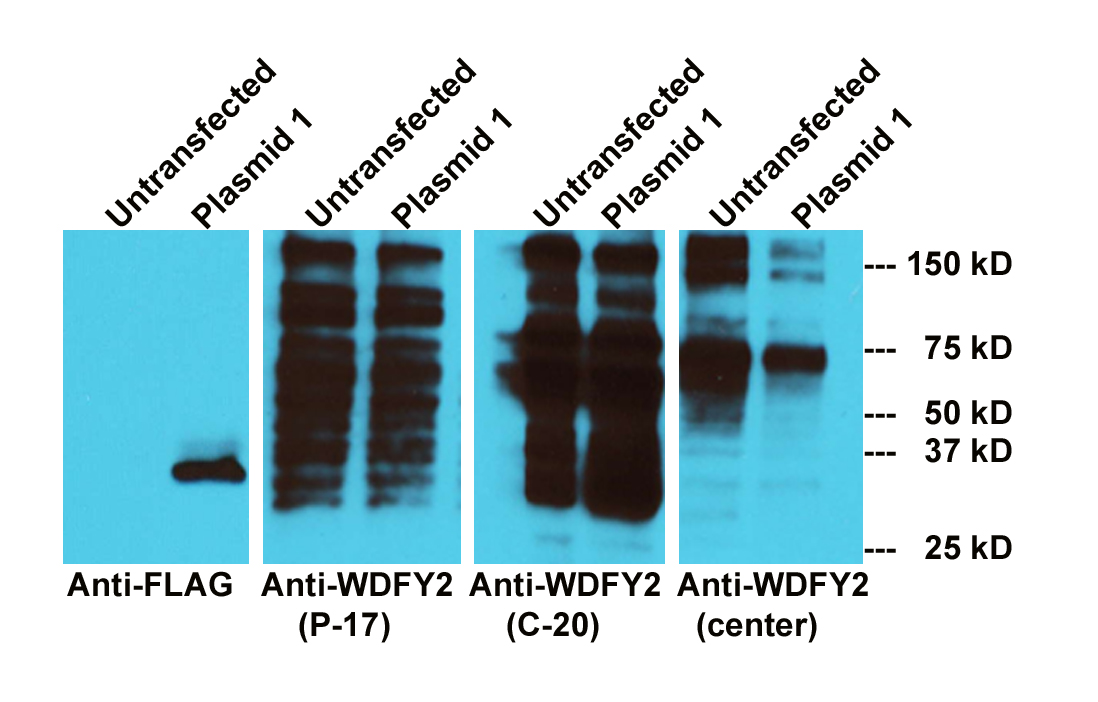
**

Supplement: Figure S3 — Commercial antibodies to WDFY2 do not recognize short WDFY2 protein isoform produced from the fusion transcript. Protein assay of untransfected HEK-293T cells and plasmid 1 transfected HEK-293T cells with the indicated antibodies. All three WDFY2 antibodies did not recognize the short WDFY2 protein isoform (as seen in the FLAG western) but recognized a number of non-specific bands leading to the conclusion that these antibodies could not be used on patient tissues. (DOCX) [file pgen.1004216.s003.docx]

**Figure S4**


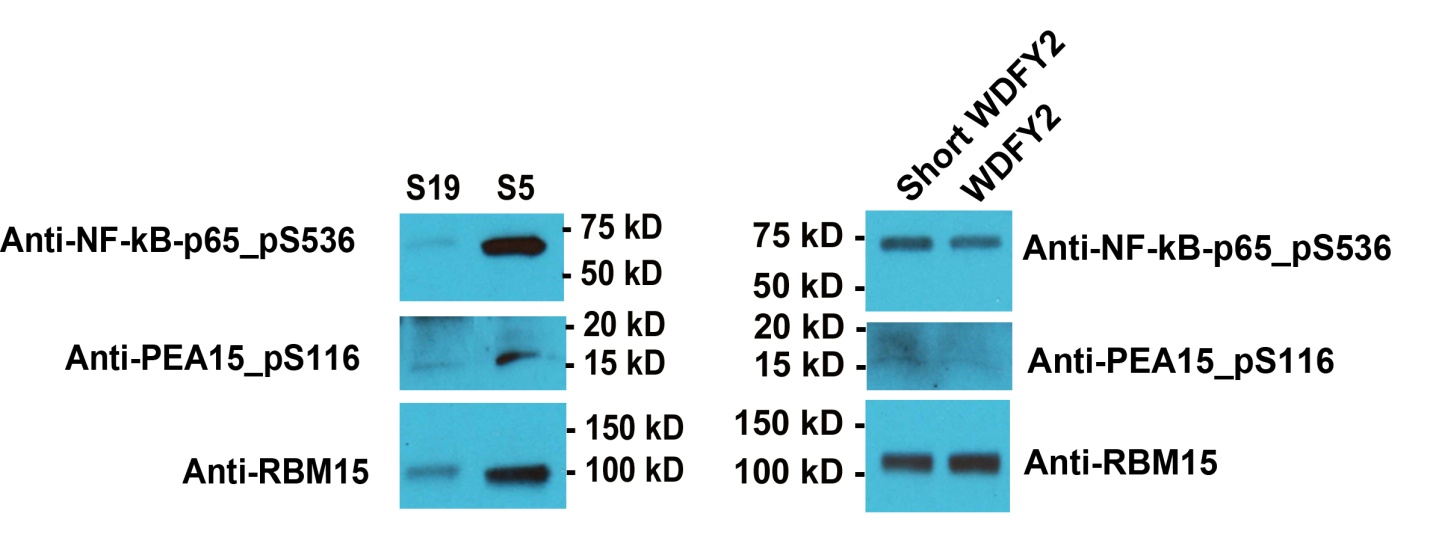

Supplement: Figure S4 — Validation of RPPA results. Western blots were performed using the indicated antibodies to confirm the results from RPPA study. Results confirmed that these three proteins are upregulated in the patient sample S5 (containing CDKN2D-WDFY2 fusion gene) as compared to sample S19 (which does not contain the fusion gene). Similarly, these proteins are upregulated in OVCAR8 transfected with short versus wildtype WDFY2. The differences between the tissue samples are more pronounced than the cell line samples, and this is consistent with the quantitative data obtained by RPPA. (DOCX) [file pgen.1004216.s004.docx]

**Figure S5**

**
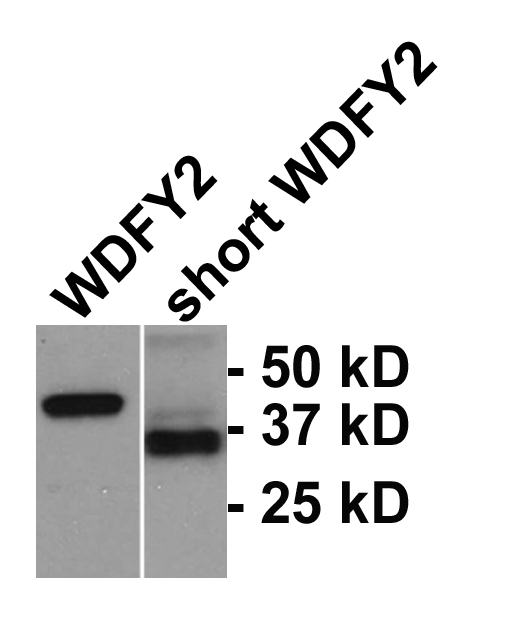
**

Supplement: Figure S5 — Controls for RPPA experiment. Western blot using FLAG antibody on OVCAR8 cells transfected with either wildtype WDFY2 or short WDFY2. Similar levels of transfected protein are observed in both cases. (DOCX) [file pgen.1004216.s005.docx]
