# Supplementary material for: CDKN2D-WDFY2 Is a Cancer-Specific Fusion Gene Recurrent in High-Grade Serous Ovarian Carcinoma
Source: PLoS Genet. 2014 Mar 27;10(3):e1004216. doi: 10.1371/journal.pgen.1004216 (PMC3967933; doi:10.1371/journal.pgen.1004216)
Supplement: Table S1 — Statistics of output reads from high-throughput transcriptome sequencing. The raw reads, usable reads, and the uniquely mapped reads that met our stringent criteria are shown, obtained for each sample by paired-end sequencing. S refers to HG-SC sample while CC refers to the clear-cell ovarian cancer sample and EC refers to endometrioid ovarian cancer sample. (DOCX) [file pgen.1004216.s006.docx]

**Table S1**

| **Patient ID** | **Sequencing read output** | **Raw reads** | | **Usable reads** | | **Unique mapped reads** | |
| --- | --- | --- | --- | --- | --- | --- | --- |
|  |  | **Read 1** | **Read 2** | **Read 1** | **Read 2** | **Read 1** | **Read 2** |
| S3 | PE 75 nts | 15700413 | 15700413 | 9082678 | 8640813 | 6258064 | 5972098 |
| S4 | PE 75 nts | 21175002 | 21175002 | 15381465 | 7674635 | 9486368 | 4664055 |
| S5 | PE 75 nts | 21233379 | 21233379 | 18243494 | 7137554 | 11912647 | 4593413 |
| S6 | PE 75 nts | 22925635 | 22925635 | 21084989 | 10381051 | 13998429 | 6893489 |
| S10 | PE 100 nts | 79371919 | 79371919 | 71940477 | 71550960 | 25235994 | 25304742 |
| S11 | PE 100 nts | 71127497 | 71127497 | 63394713 | 64725967 | 29685818 | 30327150 |
| S13 | PE 100 nts | 72589476 | 72589476 | 66182691 | 66061840 | 31231963 | 31266275 |
| Ovary pool | PE 100 nts | 69565443 | 69565443 | 63525496 | 63456240 | 32125852 | 32130238 |
| Fallopian tube pool | PE 100 nts | 196655083 | 196655083 | 132562941 | 125711733 | 90334100 | 85347045 |

**Total unique mapped reads = 476767740**

| **Patient ID** | **Sequencing read output** | **Raw reads** | | **Usable reads** | | **Unique mapped reads** | |
| --- | --- | --- | --- | --- | --- | --- | --- |
|  |  | **Read 1** | **Read 2** | **Read 1** | **Read 2** | **Read 1** | **Read 2** |
| CC2 | PE 75nts | 19724134 | 19724134 | 18421154 | 18182113 | 9547942 | 9407261 |
| EC2 | PE 75nts | 22711791 | 22711791 | 20916555 | 18382431 | 11229575 | 9829386 |
| EC4 | PE 75nts | 18704243 | 18704243 | 17547829 | 17309495 | 11314115 | 11158241 |

**Total unique mapped reads = 62486520**
