# Supplementary material for: CDKN2D-WDFY2 Is a Cancer-Specific Fusion Gene Recurrent in High-Grade Serous Ovarian Carcinoma
Source: PLoS Genet. 2014 Mar 27;10(3):e1004216. doi: 10.1371/journal.pgen.1004216 (PMC3967933; doi:10.1371/journal.pgen.1004216)
Supplement: Table S2 — Junction sequences of validated fusion transcripts in HG-SC samples. RNA junction sequences as determined by RT-PCR and Sanger sequencing are shown for each fusion transcript. The junction is denoted by an asterisk. For CDKN2D-WDFY2, the sequence of the genomic breakpoint is also shown by an asterisk. (DOCX) [file pgen.1004216.s007.docx]

**Table S2**

| **Fusion transcript** | **Chromosome** | **RNA junction sequence identified by Sanger sequencing (5'-> 3')** |
| --- | --- | --- |
| ***CDKN2D-WDFY2*** | Chr 19 & Chr 13 | GAACCGGACGTCATCCGACGCCCTCAACCGCTTCGGCAAGACGGCGCTGCAG*****CTCCATGTTCATGCATGTCTTTTAACCCGGAAACAAGAAGACTGTCCATAGGTCTAGACAATGGTACA |
| ***TMEM66-MSRB3*** | Chr 8 & Chr 12 | TGCTGACCGCGGGCCCTGCCCTGGGCTGGAACGACCCTG*****GTTGGCCTTCATTCCACGATGTGATCAATTCTGAGGCAATCACATTCACAGATGACTTTTCCTATGGGA |
| ***FAM19A3-LPP*** | Chr 1 & Chr 3 | CCGGGGGAGGAGTGTAAGGTGCTCCCGGACCTGTCGGGATGGAGCTGCAGCAGTGGACACAAAGTCAAAACCACCAAG*****TTACTCCAATAAGTCCCGCCTCTCCTTCCTCCAGTTTGGCTGGACAAGCTAAGTTTTCACAT |
| ***RFX2-CCDC94*** | Chr 19 | GGGGACCAACACGCAGCAGCCGCTGCCGCCGCCGCGGGAGCCGCTGCCCGAACTCCCGGCCCGAACTCCAG*****GGCCCTGTTGGAGGAAGCCAGAAAGCGAAGACTGCTGGAGGACTCCGACTCAGAGGATGAGGCTG |
| ***NR2F6-MAST3*** | Chr 19 | GCACCACCGGAACCAGTGCCAGTACTGCCGTCTCAAGAAGTGCTTCCGGGTGGGCATGAGGAAGGAGG*****AGATGGTGCCACTGAGTCACCTCGAAGAAGAACAGCCCCCAGCACCTGAGTCCCCAGAGAGCCGCGCCC |
| ***WDFY2-S1PR5*** | Chr 13 & Chr 19 | GTGATGGTGGGATTGTCGTCTGGAACATGGACGTGGAGAGGCAGGAG*****GACAGAGGTCTCAGCTCACATCACAAGTCACTACCTCTGCAGAGAGGTCTTTCCTGCCCACCATCACCATCTCTGTCGTTTGTCACTGGAAT |
| ***CRTAC1-GOLGA7B*** | Chr 10 | TTTCGGGTCTCAGAGTGCAGTCGGGGCTACGAGCCCAACGAGGATGGCACAGCCTGCGTGG*****GCTGGTGGAGCCCTGTGTTGAAGATAGTGACACCACAAGTTGGGAAGAGCCTTGGTCCCTGAATCACTGAATCACTG |
| ***LAMC2-NMNAT2*** | Chr 1 | CAGAAGGTGAGAACAGAAGCCCAGAAGGTTGATATCAGAGCCAAGATCGATGGGGTTGCAGTCCTAGACACTCTGACGCATTAGACGGCGTCGAGCATCTGATGG*****ACCCTGCACTTGATGACCAGCTGCACACCCGATT |
| ***MAG-CD22*** | Chr 19 | CGGCAGGGGACAACCCTCCCGTCCTGCTCAGCAGCGACTTTCGCATCTTTCGGGCACCAGAGAAGTAGG*****AGATGCTGCCAGGGTCCCTGAAGAGGGAAGACACGCGGAAACAGGTAAAAATCATTTTGCTTTTATTTTG |
| ***HSP90B1-C12orf73*** | Chr 12 | AACAGAGCAAGACGAAGATGAAGAAATGGATGTGGGAACAGATGAAGAAGAAGAAACAGCAAAG*****ATGAGCTGAGCTGGACAACTAAAAGCACCTGTAGCAGTTCTCCCCAACACAGCACTGAGCACATCTAGTAGC |
| ***SLC25A29-BC014138*** | Chr 14 | GCTCAGCAGAGAACAGTATGGGACCCCCTCACCAGGCCTGGAACACCTCCAGCCACAAAGAAGCCAAAG*****ATTAAACTCCTTACAGAAGTTAACTCTGCAGACTGAGAAGACGTGAACAGAGAGAGTCAAGGGGAGTGAT |
| ***RNF19B-BC036308*** | Chr 1 | GTGAACTAAAGCATAGTGCACGCAAGCACTCGTGCTATGGCCGGTTCCATAATCAGTTCCTACAACCCACAGGACAG*****CCTAGTATCAGAAGGCCAGGCGAGACTGCAACACTGCTCATCACCCCGCGGCGTGATCCCTG |
| ***C3orf78-PBRM1*** | Chr 3 | TAAAACGACTCGGTCGAGCATGTTCACCAGGGCCC*****AGATTCAACCTCATCTTCCCTAATTCAATGACACTCACCTGCCCA |
| ***RHOBTB2-PEBP4*** | Chr 8 | GCGAAGGCGCTGCCGCGAAGACGCCTGGCGCTGCAGTCCCAG*****GTGCCCATGGGTTGGACAATGAGGCTGTCACAGCAGCACTGTACG |
| ***KRT7-KRT86*** | Chr 12 | TCGTGGATACTGACTGGCTGGAATCGAGACGCCACCTACCGCAAGCTGCTGGAGGGCGAGGAGAGCCG*****AGTCTGCAAACTACGGCCACATGCAGGCTTCCTGTTTGTCAATCAAGTTTTCTTGGAACCCAGTCATACCG |

***CDKN2D-WDFY2* genomic breakpoint sequence:**

ttagcatcttctctgggaggattcctggttctctgagtgccggggattcactttccctggggaggttcttattccgtgggtgcgggttcagctcctttggatgccgtttccatggggacggctcacgtttcttggggcttctatggggagatccgatctctggaaggagaggtcctgtctctaggggttcctcattttgtagggggctctctgagacaaatctggttacttgggggtggggtcttcattcctaaaggttatgcggatccttgatatagtatgggggccccaatggaggaacaatgtcagattgcaagacatccctatattccctgggagacattttacagggaagtctcgtttctcttttattattacttttggaatcccgcttcttgaaaggagctctcaggtactctgaggtgacatcaccattatgtaggaggaggctatttcctggagaaggggttcgctt*****gattgctggatcaaatggtagttctacttttagttatttaaggaatctccacactgttttccatagtggttgtactagtttgcattcccaccagcagcgcagaggtgttcccttttcgccacatccatgccaacatctattatattttgattttttgattatggccattcttgcaggagtaaggtgataccgcattgcagttttgatttgcatttccctgatcattagtgatgttgagcgatttttcaagtttgttggccatttgtatatcttttgagaattgtctattcatgtccttagcccactttttgatggggttctttttgtgtgtgtgtgctaatttgtttgagttccttgtagattctgcatattagtcctttgttggatgttttaattgtttgttgtgtgcgttaattgctaagatttttttccca
