# Supplementary material for: CDKN2D-WDFY2 Is a Cancer-Specific Fusion Gene Recurrent in High-Grade Serous Ovarian Carcinoma
Source: PLoS Genet. 2014 Mar 27;10(3):e1004216. doi: 10.1371/journal.pgen.1004216 (PMC3967933; doi:10.1371/journal.pgen.1004216)
Supplement: Table S3 — Paired chimeric reads and junction reads supporting fusion transcripts from each patient sample. The number of paired chimeric reads and junction reads obtained for each of the 15 fusion transcripts contributed by each sequenced sample is shown. For example, “7+19” indicates 7 paired chimeric reads and nineteen junction reads for the fusion transcript FAM19A3-LPP in sample S3. (DOCX) [file pgen.1004216.s008.docx]

**Table S3**

| **Fusion Transcript** | **S3** | **S4** | **S5** | **S6** | **S10** | **S11** | **S13** | **Ovary pool** | **Fallopian tube pool** | **Total reads** |
| --- | --- | --- | --- | --- | --- | --- | --- | --- | --- | --- |
| *CDKN2D-WDFY2* | 0 | 0 | 3+7 | 0 | 0 | 0 | 0 | 0 | 0 | 10 |
| *TMEM66-MSRB3* | 0 | 0 | 0 | 6+5 | 0 | 0 | 0 | 0 | 0 | 11 |
| *FAM19A3-LPP* | 7+19 | 0 | 0 | 0 | 0 | 0 | 0 | 0 | 0 | 26 |
| *RFX2-CCDC94* | 0 | 0 | 8+3 | 0 | 0 | 0 | 0 | 0 | 0 | 11 |
| *NR2F6-MAST3* | 0 | 3+7 | 0 | 0 | 0 | 0 | 0 | 0 | 0 | 10 |
| *WDFY2-S1PR5* | 0 | 0 | 5+10 | 0 | 0 | 0 | 0 | 0 | 0 | 15 |
| *CRTAC1-GOLGA7B* | 0 | 11+20 | 0 | 0 | 0 | 0 | 0 | 0 | 0 | 31 |
| *LAMC2-NMNAT2* | 9+0 | 4+0 | 4+0 | 0 | 1+0 | 0 | 0 | 0 | 1+0 | 19 |
| *MAG-CD22* | 0 | 0 | 2+0 | 7+15 | 0 | 0 | 0 | 1+0 | 0 | 25 |
| *HSP90B1-C12orf73* | 0 | 2+0 | 0 | 0 | 0 | 0 | 0 | 1+0 | 1+0 | 4 |
| *SLC25A29-BC014138* | 0 | 5+6 | 0 | 0 | 0 | 0 | 0 | 0 | 0 | 11 |
| *RNF19B- BC036308* | 2+15 | 0 | 0 | 0 | 0 | 0 | 0 | 0 | 0 | 17 |
| *C3orf78-PBRM1* | 0 | 3+0 | 0 | 0 | 0 | 0 | 0 | 0 | 0 | 3 |
| *RHOBTB2-PEBP4* | 0 | 3+1 | 0 | 0 | 0 | 0 | 0 | 0 | 0 | 4 |
| *KRT7-KRT86* | 4+0 | 5+2 | 2+0 | 0 | 0 | 0 | 0 | 0 | 0 | 13 |
