# Supplementary material for: CDKN2D-WDFY2 Is a Cancer-Specific Fusion Gene Recurrent in High-Grade Serous Ovarian Carcinoma
Source: PLoS Genet. 2014 Mar 27;10(3):e1004216. doi: 10.1371/journal.pgen.1004216 (PMC3967933; doi:10.1371/journal.pgen.1004216)
Supplement: Table S4 — Primers used in experiments. (DOCX) [file pgen.1004216.s009.docx]

**Table S4**

| **Primers for Validation and Nested RT-PCR of fusion transcripts** | **Sequence 5'-> 3'** |
| --- | --- |
| CDKN2D-WDFY2 F5 | CGACATGCTGCTGGAGGAGGT |
| CDKN2D-WDFY2 F1 | GCAGGAGGTGCGCCGCCTTCT |
| CDKN2D-WDFY2 R1 | GTACCATTGTCTAGACCTATGGA |
| TMEM66-MSRB3 F1 | CCCATAGGAAAAGTCATCTGT |
| TMEM66-MSRB3 R1 | CTGCTTGCTCCTCGGCTTGCA |
| TMEM66-MSRB3 R2 | GCTTGCATTTGTTTCTGCTGA |
| FAM19A3-LPP F1 | AATGGACAAGTTTCTCCTTCCCCA |
| FAM19A3-LPP R1 | CACAGCCTCCATCGTCCTGCA |
| FAM19A3-LPP F2 | GGAGAGGCGGGACTTATTGGA |
| RFX2-CCDC94 F1 | CATAGAGACTGTAGCCGTGGA |
| RFX2-CCDC94 R1 | TTACCTCATCCAGGATGGCGGT |
| RFX2-CCDC94 F2 | GACCAACACGCAGCAGCCGCT |
| RFX2-CCDC94 R2 | GCTTTCTGGCTTCCTCCAACA |
| NR2F6-MAST3 F1 | GCAAGCATTACGGTGTCTTCA |
| NR2F6-MAST3 R1 | GTTACGCAGGATCAAGTTCTGT |
| NR2F6-MAST3 F2 | AACCTCAGCTACACCTGCCGGT |
| NR2F6-MAST3 R2 | CAAAGTCGCTTTCGCATGGCT |
| WDFY2-S1PR5 F1 | CACAGAAAAGAACTCGGTGATGA |
| WDFY2-S1PR5 R1 | TCTTTCACAGCGACAGAGTCCA |
| CRTAC1-GOLGA7B F1 | GTTCCAGGTAAGTGTTCCTGA |
| CRTAC1- GOLGA7B R1 | GTATGTGTCAACACCTATGGA |
| LAMC2-NMNAT2 F1 | AGGAGCTGGAGTTTGACACGA |
| LAMC2-NMNAT2 R1 | CGCAAATTCAAATAAGCCAGT |
| MAG-CD22 F1 | CCATCCTGATTGCCATCGTCT |
| MAG-CD22 R1 | CCAGCTCTGGGAGGTATGCCT |
| HSP90B1-C12orf73 F1 | GTCGAGCAGAGGAGACCATGA |
| HSP90B1-C12orf73 R1 | ACAGAGCAAGACGAAGATGAAGA |
| SLC25A29-BC014138 F1 | CTTGATCCCTTGGGGGTGCCTT |
| SLC25A29-BC014138 R1 | AAGTGTGCAGAGGGCTCAGCA |
| RNF19B-BC036308 F1 | GGAAATTTTCCCCAAAGACACA |
| RNF19B-BC036308 R1 | CTCACCATCCTCACCCTGACCA |
| C3orf78-PBRM1 F1 | ATCCAGTGCTTAGTTCCGTCA |
| C3orf78-PBRM1 R1 | TGGGCAGGTGAGTGTCATTGA |
| RHOBTB2-PEBP4 F1 | GGGCTGTTCTCATCCTCGTCT |
| RHOBTB2-PEBP4 R1 | GCGAAGGCGCTGCCGCGAAGA |
| KRT7-KRT86 F1 | CTGCGTGAGTACCAGGAACTCA |
| KRT7-KRT86 R1 | ATACTTGAAGGAAGCGGTATGA |
|  |  |
| **Long range PCR** |  |
| CDKN2D-WDFY2 F1 | GCAGGAGGTGCGCCGCCTTCT |
| CDKN2D-WDFY2 R2 | ACAACACTGCATAGGCTACTCT |
| CDKN2D-WDFY2 R3 | ACACTGGGTACACTGGTCGGGT |
| CDKN2D-WDFY2 R4 | GCATTATTTGGCAGGAGACACT |
| CDKN2D-WDFY2 R5 | TAGGGCTACTGCTCCCAATCT |
| CDKN2D-WDFY2 R6 | AGAACACAGCCAGGATTCTCA |
| CDKN2D-WDFY2 R7 | AGACATGCAAAGCCTCAAAGA |
|  |  |
| **Primers for CDKN2D- WDFY2-FLAG construct:** |  |
| CDKN2D CDS fwd | TCGACATGCTGCTGGAGGAGGT |
| WDFY2 exon 11 rev | TAACCTTGTCAGTTCCAGAAGT |
| Updated E-S-N primer | GGCAAAGAATTCATAATTAACCGCGGGCGGCCGCCATGCT |
| Bam-Stop-FLAG-WDFY2 CDS end rev | GCGGATCCCGTCATCACTTGTCGTCATCGTCTTTGTAGTCAGACACGACTGGGGTCA |
|  |  |
| **Primers for Truncated CDKN2D-FLAG construct:** |  |
| W-C F5 | CGACATGCTGCTGGAGGAGGT |
| W-C R4 | ACAGTCTTCTTGTTTCCGGGT |
| Eco-Sac-Not-Kozak-CDKN2D fwd | AAGAATTCCGCGGGCGGCCGCCATGCTGCTGGAGGAGGTTCG |
| Bam-Stop-FLAG WDFY2 rev | GCGGATCCCGTTACTACTTGTCGTCATCGTCTTTGTAGTCTGGACAGTCTTCTTGTTT |
|  |  |
| **Primers for WDFY2-FLAG construct:** |  |
| WDFY2 CDS fwd | ATGGCGGCGGAGATCCAGCCCA |
| WDFY2 end rev | TCAAGACACGACTGGGGTCA |
| Updated E-S-N primer for WDFY2 | GGCAAAGAATTCATAATTAACCGCGGGCGGCCGCCATGGC |
| Bam-Stop-FLAG-WDFY2 CDS end rev | GCGGATCCCGTCATCACTTGTCGTCATCGTCTTTGTAGTCAGACACGACTGGGGTCA |
|  |  |
| **Primers for short WDFY2-FLAG construct:** |  |
| Updated E-S-N primer for short WDFY2 | GGCAAAGAATTCATAATTAACCGCGGGCGGCCGCCATGTCT |
| Bam-Stop-FLAG-WDFY2 CDS end rev | GCGGATCCCGTCATCACTTGTCGTCATCGTCTTTGTAGTCAGACACGACTGGGGTCA |
