# Supplementary material for: CDKN2D-WDFY2 Is a Cancer-Specific Fusion Gene Recurrent in High-Grade Serous Ovarian Carcinoma
Source: PLoS Genet. 2014 Mar 27;10(3):e1004216. doi: 10.1371/journal.pgen.1004216 (PMC3967933; doi:10.1371/journal.pgen.1004216)
Supplement: Table S5 — Antibodies used in RPPA experiments. (DOCX) [file pgen.1004216.s010.docx]

**Table S5**

| ACC1 | eEF2K | p38_MAPK | Rictor |
| --- | --- | --- | --- |
| ACC_pS79 | EGFR | p38_pT180_Y182 | Rictor_pT1135 |
| ACVRL1 | EGFR_pY1068 | JNK_pT183_pY185 | S6_pS235_S236 |
| Akt | EGFR_pY1173 | JNK2 | S6_pS240_S244 |
| PRAS40_pT246 | eIF4E | c-Met_pY1235 | p90RSK |
| Akt_pS473 | 4E-BP1 | c-Myc | p90RSK_pT359_S363 |
| Akt_pT308 | 4E-BP1_pS65 | MYH11 | p70S6K |
| Annexin_VII | 4E-BP1_pT37_T46 | Myosin IIa pS1943 | p70S6K_pT389 |
| AR | eIF4G | NDRG1_pT346 | Raptor |
| Bad_pS112 | HER2 | NF2 | SCD1 |
| Bak | HER2_pY1248 | NF-kB-p65_pS536 | SF2 |
| Bax | HER3 | Notch1 | Smad1 |
| Bcl-2 | HER3_pY1289 | N-Ras | Smad3 |
| Bcl-xL | MIG-6 | Heregulin | Smad4 |
| Bim | ER-alpha | DJ-1 | Src |
| Beclin | ER-alpha_pS118 | PCNA | Src_pY416 |
| Bid | FASN | PDCD4 | Src_pY527 |
| cIAP | Fibronectin | PDK1 | STAT3_pY705 |
| B-Raf | FoxM1 | PDK1_pS241 | STAT5-alpha |
| BRCA2 | FOX03a | PEA15 | Stathmin |
| TIGAR | FOXO3a_pS318_S321 | PEA15_pS116 | Syk |
| Caspase7_cleavedD198 | mTOR | CD31 | Transglutaminase |
| Caveolin-1 | mTOR_pS2448 | PR | p53 |
| Cyclin_B1 | G6PD | PI3K-p110-alpha | 53BP1 |
| Cyclin_D1 | Gab2 | PI3K-p85 | TRFC |
| Cyclin_E1 | GAPDH | PKC-pan_BetaII_pS660 | TSC1 |
| CDK1 | GATA3 | AMPK_alpha | Tuberin |
| E-Cadherin | GSK3_pS9 | AMPK_pT172 | Tuberin_pT1462 |
| N-Cadherin | GSK3-alpha-beta_pS21_S9 | PKC-alpha | TTF1 |
| p21 | GSK3-alpha-beta | PKC-alpha_pS657 | VHL |
| p27 | IGFBP2 | PKC-delta_pS664 | TAZ |
| p27_pT157 | INPP4B | PTEN | XRCC1 |
| p27_pT198 | IRS1 | Paxillin | YAP |
| Chk1 | CD49b | Rab11 | YAP_pS127 |
| Chk1_pS345 | c-Jun_pS73 | Rab25 | YB-1 |
| Chk2 | VEGFR2 | Rad50 | YB-1_pS102 |
| Chk2_pT68 | c-Kit | Rad51 | 14-3-3_beta |
| Claudin-7 | Lck | C-Raf | 14-3-3_epsilon |
| Collagen_VI | MEK1 | C-Raf_pS338 | 14-3-3_zeta |
| beta-Catenin | MEK1_pS217_S221 | Rb_pS807_S811 | RBM15 |
| eEF2 | MAPK_pT202_Y204 |  |  |
